# Asymptomatic Gastric Giant Polyp in a Boy with Peutz-Jeghers Syndrome Presented with Multiple Café Au Lait Traits

**DOI:** 10.1155/2018/6895974

**Published:** 2018-09-19

**Authors:** Christos Plataras, Efstratios Christianakis, Florentia Fostira, George Bourikis, Maria Chorti, Dimitrios Bourikas, Nikolaos Fotopoulos, Konstantinos Damalas, Khalil Eirekat

**Affiliations:** ^1^Pediatric Surgery Department, Penteli Children's Hospital, Ippokratous 8, Penteli 15236, Greece; ^2^Department of Genetics, Demokritos Research Center, Neapoleos 10, Agia Paraskevi 153 10, Greece; ^3^General Surgery Department, Tzanio General Hospital, Leoforos Afentouli ke Zanni, Piraeus 185 36, Greece; ^4^Histopathology Department, Sismanoglio General Hospital, Sismanogliou 37, Marousi 151 26, Greece; ^5^General Surgery Department, Sismanoglio General Hospital, Sismanogliou 37, Marousi 15126, Greece; ^6^General Surgery Department, Agios Savvas Regional Cancer Hospital, Leof. Alexandras 171, Athens 11522, Greece

## Abstract

We describe an asymptomatic case of PJS in a six-year-old boy with café au lait spots in several parts of his body, a large gastroduodenal polyp, two polyps near the ampulla of Vater, and another in the jejunum. This patient shows some unique aspects of PJS. No other such large gastric polyp in a Peutz-Jeghers child is reported in the literature. The large size of the gastric polyp with lack of symptoms is unusual and poses a unique challenge in terms of management and surgical resection.

## 1. Introduction

Peutz-Jeghers syndrome (PJS) is an autosomal dominant disorder distinguished by hamartomatous polyps in the gastrointestinal tract and pigmented mucocutaneous lesions. Coexistence of multiple café au lait spots is rare [1, 2].

We describe an asymptomatic case of PJS in a six-year-old boy with café au lait spots in several parts of his body, a large gastroduodenal polyp, two polyps near the ampulla of Vater, and another in the jejunum.

## 2. Case Presentation

An asymptomatic 6-year-old boy was referred by a dermatologist because of lesions on the inner side of his lower lip that firstly appeared 4 years ago.

**Figure 1:**
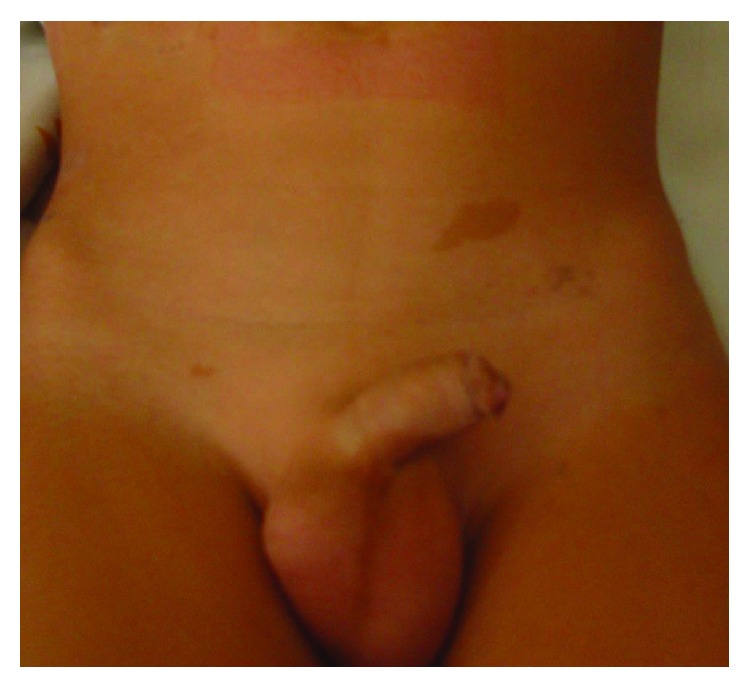
Skin lesions resembling café au lait spots.

He was a skinny boy, light-coloured skin, blond, and green-eyed that was always eating small meals. He had no previous family history of PJS. On clinical examination, we found seventeen café au lait spots ranging from 0.3–3 cm on the anterior and posterior body surface and extremities (Figure 1).

**Figure 2:**
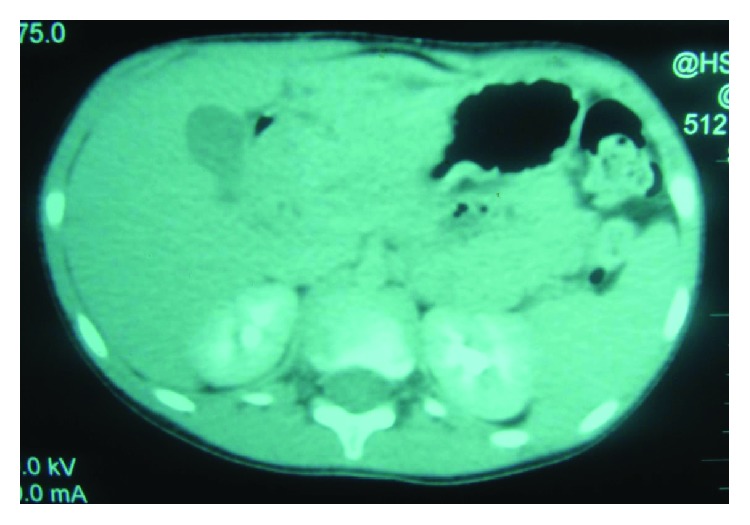
Abdominal CT scan. Α large polypoid gastric mass in the antrum and the beginning of the duodenum.

Blood tests showed mild anemia. Abdominal ultrasound and computed tomography showed a large polypoid gastric mass in the antrum and the beginning of the duodenum (Figure 2).

A large, 8 × 5 cm in size, multilobed polypoid gastric mass situated in the antrum was found in gastroscopy. The mass was hemorrhagic, wide-based, and seemed to enter duodenum but moved back to the antrum with peristaltic movements. Two smaller polyps, 0.5 cm in size, were found at the 2nd part of the duodenum near the ampulla of Vater.

**Figure 3:**
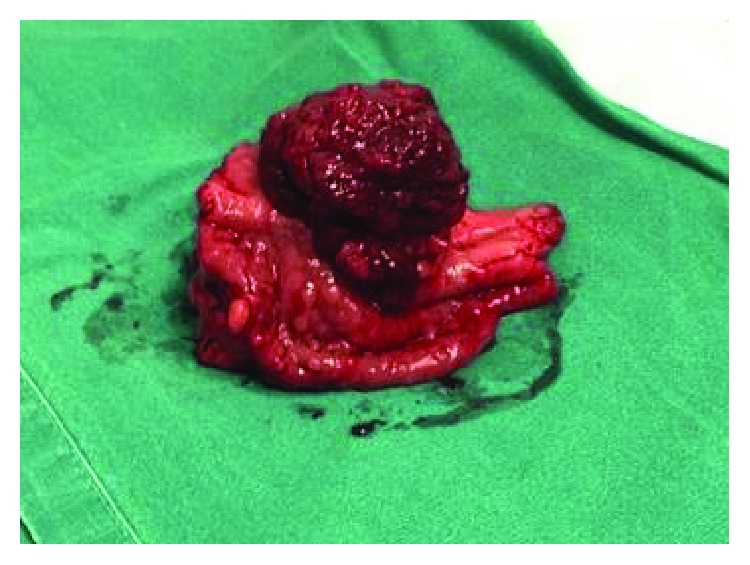
Macroscopic appearance of the polyp after surgical removal.

The operation was scheduled for polyp removal. Under general anesthesia, a hard epigastric mass was palpated. We made a midline supraumbilical incision. The hard mass could be palpated at the lower third of the stomach. Palpation also revealed one lesion at the second part of the duodenum and another in the jejunum. We did a gastrotomy on the anterior surface of the pyloric antrum. The polyp was wide-based (Figure 3), occluding almost completely the pylorus and the duodenum only leaving a space for a hand's little finger to pass. We proceeded to a lower third gastrectomy involving the duodenal bulb, pylorus, and antrum and performed a Billroth I anastomosis. We also did a longitudinal incision of the jejunum 15 cm away from the ligament of Treitz and managed to remove one wide-based polyp, which is 1.5 cm in length. His postoperative course was uneventful.

STK11/LKB1 gene identification (a gene encoding a serine/threonine kinase that is responsible for the appearance of the syndrome) (1, 2) showed the splicing mutation: c290 + 1 G > A, in intron 1, which results in aberrant splicing. No family history was reported, so it is highly likely that this mutation was a de novo event. This could not be confirmed, as the patient's parents did not consent to genetic testing.

Postoperative results were excellent. We advised for clinical examination and ultrasound of testis yearly and capsule endoscopy, colonoscopy, and gastroscopy biannually.

**Figure 4:**
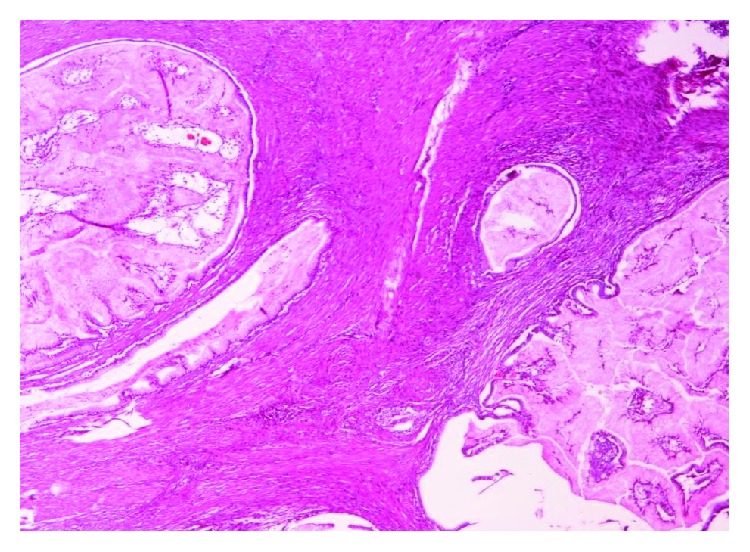
Histological appearance of the gastric polyp.

At a second look gastroscopy, six months later, we managed to endoscopically remove two smaller polyps near the ampulla of Vater. Colonoscopy was clear. Histology verified the diagnosis of PJS. All 4 polyps showed findings suggestive of hamartomas (Figure 4).

So far, one polyp was detected in the duodenum and another in the sigmoid colon. Polyps were removed by gastroscopy and colonoscopy, respectively. The duodenal polyp was found to be histologically a Peutz-Jeghers polyp, whereas sigmoid polyp was found to be of a hyperplastic type. The gastric polyp was also found to have abnormal (resembling neoplastic) growth in muscularis mucosae where smooth muscle fibers followed the growth of exophytic gastric pits. Biopsies of the esophagus were normal, and gastric biopsies revealed only signs of mild chronic gastritis.

## 3. Discussion

Primary tumors of the gastrointestinal tract in children are estimated at 1.2% of all pediatric malignancies. They are usually benign. The literature is mainly limited to case studies [3, 4].

The prevalence of gastric polyps in the pediatric population is low compared with that in adults (0.7% vs. 6.35%). The most common pathological types in children are hyperplastic polyps, and most are asymptomatic and do not require removal. Gastric polyps are being encountered in less than 1% of upper gastrointestinal endoscopies performed in children [5, 6].

PJS is an autosomal dominant disease characterized by mucocutaneous pigmentation and hamartomatous polyps of the gastrointestinal tract. The most common location of these polyps is in the small intestine, colon, and stomach, respectively [1, 2].

Our patient shows some unique aspects of PJS. At first, the large size of the gastric polyp with lack of symptoms is unusual and poses a unique challenge in terms of management and surgical resection. No other such large gastric polyp in a Peutz-Jeghers child is reported in the literature. Polypectomy or partial resection was impossible due to its size. The polyp obstructed the view and precluded initial endoscopy of the duodenum. Resection was the only way to inspect the duodenum and first part of the jejunum. Moreover, a Billroth I anastomosis was deemed preferable so that future endoscopies may be facilitated.

Multiple café au lait spots on his body in addition to the classic mucocutaneous macules around the mouth and the buccal mucosa was another unusual finding. Café au lait spots are more often associated with syndromes such as neurofibromatosis type 1 and McCune-Albright syndrome [1, 2]. Unfortunately, skin lesions on our patient were not given attention from his parents and community pediatrician. He was referred for dermatology evaluation many years after the first appearance. So it is important to stress that both unusual and classic hyperpigmentation in children could be the sign of a syndrome that may be “clinically silent” and may have important clinical implications (e.g., in terms of screening). Clinicians should be aware and refer in time.

Surveillance of PJS patients is of critical importance because of the increased risk for malignancies [7, 8]. We advised for clinical examination of testis yearly and capsule endoscopy, colonoscopy, and gastroscopy every three years.

Polyps beyond the reach of conventional endoscopy have been difficult to manage. Until recently, barium contrast upper-gastrointestinal series with a small-bowel follow-through has been recommended. However, recent advances allow better diagnosis and eradication of small-bowel polyps. Video capsule endoscopy (VCE), magnetic resonance enterography (MRE), and double balloon enteroscopy (DBE) are the modern ways of small bowel surveillance [8]. In our patient, we chose VCE due to its availability in our hospital. So far, no malignancy has been found, and the patient remains in excellent clinical condition.

### 3.1. Summary Box

- PJS can present with café au lait traits and hyperpigmented macules of lips
- Unusual and classic hyperpigmentation in children may indicate a “clinically silent” syndrome
- Peutz-Jeghers gastric polyps in children are very rare
- They may become very large in size in small children but still be asymptomatic
- Prompt diagnosis may avoid complicated operations and prevent any possibility for malignant transformation
- Follow-up of children with PJS is necessary for early diagnosis and management of possible tumors
